# S1PR2 antagonist ameliorate high glucose-induced fission and dysfunction of mitochondria in HRGECs via regulating ROCK1

**DOI:** 10.1186/s12882-019-1323-0

**Published:** 2019-04-18

**Authors:** Wei Chen, Hong Xiang, Ruifang Chen, Jie Yang, Xiaoping Yang, Jianda Zhou, Hengdao Liu, Shaoli Zhao, Jie Xiao, Pan Chen, Alex F. Chen, Shuhua Chen, Hongwei Lu

**Affiliations:** 1 Center for Experimental Medical Research, The Third Xiangya Hospital of Central South University, 138 Tongzipo Road, Changsha, Hunan 410013 People’s Republic of China; 2 Department of Cardiology, the Third Xiangya Hospital of Central South University, Changsha, Hunan 410013 People’s Republic of China; 3 Department of Nursing, School of Medicine, Hunan Normal University, Changsha, Hunan 410013 People’s Republic of China; 4 Department of Pharmacy, School of Medicine, Hunan Normal University, Changsha, Hunan 410013 People’s Republic of China; 5 Department of Burn, The Third Xiangya Hospital of Central South University, Changsha, Hunan 410013 People’s Republic of China; 6 Department of Surgery, University of Pittsburgh School of Medicine, Pittsburgh, PA 15213 USA; 7 Department of Biochemistry, School of Life Sciences of Central South University, 138 Tongzipo Road, Changsha, Hunan 410013 People’s Republic of China

**Keywords:** Sphingosine-1-phosphate receptor 2, Diabetes, Mitochondria, Endothelial cell

## Abstract

**Aims:**

Sphingosine-1-phosphate receptor 2 (S1PR2) is a G-protein-coupled receptor that regulates sphingosine-1-phosphate-triggered cellular response. However, the role of S1PR2 in diabetes-induced glomerular endothelial cell dysfunction remains unclear. This study aims to investigate the effect of S1PR2 blockade on the morphology and function of mitochondria in human renal glomerular endothelial cells (HRGECs).

**Methods:**

HRGECs were pretreated with a S1PR2 antagonist (JTE-013) or a Rho-associated coiled coil-containing protein kinase 1 (ROCK1) inhibitor (Y27632) for 30 min and then cultured with normal glucose (5.5 mM) or high glucose (30 mM) for 72 h. The protein expression levels of RhoA, ROCK1, and Dynmin-related protein-1(Drp1) were evaluated by immunoblotting; mitochondrial morphology was observed by electron microscopy; intracellular levels of ATP, ROS, and Ca^2+^ were measured by ATPlite, DCF-DA, and Rhod-2 AM assays, respectively. Additionally, the permeability, apoptosis, and migration of cells were determined to evaluate the effects of S1PR2 and ROCK1 inhibition on high glucose-induced endothelial dysfunction.

**Results:**

High glucose induced mitochondrial fission and dysfunction, indicated by increased mitochondrial fragmentation, ROS generation, and calcium overload but decreased ATP production. High glucose also induced endothelial cell dysfunction, indicated by increased permeability and apoptosis but decreased migration. However, inhibition of either S1PR2 or ROCK1 almost completely blocked these high glucose-mediated cellular responses. Furthermore, inhibiting S1PR2 resulted in the deceased expression of RhoA, ROCK1, and Drp1 while inhibiting ROCK1 led to the downregulated expression of Drp1.

**Conclusions:**

S1PR2 antagonist modulates the morphology and function of mitochondria in HRGECs via the positive regulation of the RhoA/ROCK1/Drp1 signaling pathway, suggesting that the S1PR2/ROCK1 pathway may play a crucial role in high glucose milieu.

**Electronic supplementary material:**

The online version of this article (10.1186/s12882-019-1323-0) contains supplementary material, which is available to authorized users.

## Introduction

Sphingosine-1-phosphate receptors belong to G-protein-coupled receptors that participate in different cellular responses depending on cell types and availabilities of downstream effectors [1]. Sphingosine-1-phosphate receptor 2 (S1PR2) is predominately expressed in vascular tissues [2], where its activity is tightly associated with the function of vasculature, especially the function of endothelial cells [3]. JTE-013, a potent and selective S1PR2 antagonist, could reverses the inhibitory effects of S1PR2 on vascular endothelial cells [4]. Windh and colleagues have revealed that S1PR2 couples to Gi and G12/13 along with the Gq family members [5], thus activating multiple small GTPases (Rho, Rac, and Ras) [6–8] to exerts distinct effects via mediating diverse signaling pathways [9, 10]. Recently, S1PR2 has been found to be an endothelial barrier-unstabilizing mediator in the activation of Rho-associated coiled coil-containing protein kinase (ROCK) pathway [11]. Human renal glomerular endothelial cells (HRGECs) are highly specialized primary endothelial cells packed with fenestrae, which contribute to the glomerular filtration barrier [12, 13]. However, the specific role of S1PR2 in the regulatory function of HRGECs remains unclear.

As a major energy source of cells, mitochondria have an important role in the regulation of endothelial function [14]. In renal glomerular endothelial cells, mitochondria are the most important effectors of cellular metabolism and oxidative stress, and their changes in morphology and function are closely related to the varying cellular microenvironment and functions [15]. Numerous studies have shown that sphingosine-1-phosphate receptors affect the morphology and function of mitochondria in various cell types [16–19]. However, the role of S1PR2 activity in the morphology and function of mitochondria of HRGECs and the downstream signaling molecules of S1PR2 coupling associated with mitochondrial and endothelial dysfunctions have not been identified.

Endothelial dysfunction induced by high glucose (HG) is a classical in vitro model to mimic the development of diabetes. HG induces endothelial cell apoptosis, oxidative stress, and inflammation [20]. Also, HG increases permeability [21] and enhances endothelial-to-mesenchymal transition in HRGECs [22]. Moreover, increased mitochondrial fragmentation and superoxide production [23] and lowered mitochondrial bioenergetics [24] have been observed in HG-treated endothelial cells. Although mitochondrial impairment is indicative of HG-induced endothelial dysfunction, the changes of mitochondrial morphology and functions induced by HG in HRGECs and the molecular mechanisms underlying these pathological events are still unclear. In the present study, we aim to investigate whether S1PR2 modulates the morphology and function of mitochondria in HRGECs via regulating ROCK1, thus affecting the dysfunction of glomerular endothelium mediated by HG.

## Materials and methods

### Cell culture and treatment

Primary HRGECs were purchased from Sciencell (Carlsbad, CA, USA) and cultured in Endothelial Cell Medium (ECM) (Carlsbad, CA, USA) containing 5% fetal bovine serum at 37 °C in a 5% CO_2_ incubator. Cells at passages 2–4 were treated with 30 mM HG for 72 h to induce cell dysfunction [25] or treated in parallel with 5.5 mM normal glucose (NG) as a control group or 24.5 mM mannitol (Mnt) plus 5.5 mM glucose as an osmotic control. As indicated elsewhere [26], cells were pretreated with 10 μM JTE-013 (Cayman, Ann Arbor, MI, USA), a specific S1PR2 inhibitor, or 10 μM Y27632 (Selleck, Houston, TX, USA), a selective ROCK1 inhibitor, for 30 min to assess the regulatory effects of SIPR2 and ROCK1 in HRGECs.

### Immunoblotting

Total protein extracts (30 μg) was separated on 8–12% Bis-Tris gels, followed by transferring onto polyvinylidene fluoride membranes. After blocking with 5% skimmed milk for 2 h at room temperature (RT), the blots were incubated with a primary antibody overnight at 4 °C and then with horseradish peroxidase-conjugated secondary antibody for 1 h at RT. The primary antibodies included ROCK1 (Abcam, Cambridge, UK; 1:1000), RhoA (Abcam; 1:1000), and dynmin-related protein-1(Drp1) (Abcam; 1:1000). The intensities of protein bands were visualized using chemiluminescence with an ImageQuant 350 (GE Healthcare, Woburn, MA, USA). GAPDH was used as a loading control.

### Mitochondrial morphology assay

After treated with glucose at different levels, 1 × 10^6^ cells were fixed in 3% glutaraldehyde for 48 h at 4 °C and post-fixed in 1% osmium tetroxide for 2 h at RT. After pre-stained with acetatebarbitone for 10 min, dehydrated with acetone, and embedded in Epon 812, cells were isolated on nickel fitters, stained with 2% uranyl acetate for 10 min, and then with Reynold’s lead citrate for 5 min. Finally, mitochondrial morphology of HRGECs was evaluated by transmission electron microscopy (Hitachi-7650, Tokyo, Japan) at 60 kV.

### ATP assay

Intracellular ATP levels of HRGECs were quantified by using an ATPlite assay kit according to the manufacturer’s instructions (PerkinElmer, Akron, OH, USA). Briefly, following cell exposure to HG with or without pretreatment with an inhibitor, mammalian cell lysis solution was added to cell-containing 96-well microplates. After shaking for 5 min at RT, equal amount of substrate was added and mixed for another 5 min at RT. The luminescent intensities were monitored 10 min later to indicate the ATP levels and mitochondrial activities.

### Intracellular ROS assay

Intracellular ROS production of living HRGECs was detected by using 2′,7′- dichlorofluorescein diacetate (DCF-DA) (Beyotime, Jiangsu, China), which is oxidized to a fluorescent dye when ROS are present. After cultured with HG, cells were stained with 10 μM DCF-DA dye in the dark for 30 min at 37 °C and then washed twice. Intracellular fluorescence generated was subsequently imaged using a fluorescent microscope with a FITC filter (Ex/EM = 485/535 nm; Nikon, Tokyo, Japan) and quantified by a flow cytometry (Becton-Dickinson, San Jose, CA, USA).

### Intracellular calcium assay

Intracellular Ca^2+^ concentrations were detected by the Rhod-2 AM assay (Thermo Fisher, Waltham, MA, USA). After cultured with HG, cells were stained with 10 μM Rhod-2 AM dye in the dark for 30 min at 37 °C and then washed twice, and mitochondrial Ca^2+^ levels were measured by using a fluorescent microscope with filter (Ex/Em = 552/581 nm) and a flow cytometry.

### Permeability assay

Endothelial cell permeability was detected by using an In Vitro Vascular Permeability Assay kit following the manufacturer’s protocol (Millipore, Burlington, MA, USA). After cells formed a tighter monolayer and exposed to HG, they were stained with FITC-labeled BSA for 20 min, and the amount of FITC-BSA diffusion across endothelial monolayer was measured with a fluorescent microscope with a FITC filter and quantified by a fluorescence plate reader.

### Apoptosis assay

To quantify the apoptosis of HRGECs, cells were stained with Annexin V and propidium iodide (PI) using an Annexin V- FITC apoptosis detection kit (BD Biosciences, Franklin Lakes, NJ, USA) and analyzed by flow cytometry.

### Transwell migration assay

To determine the migration of HRGECs, Matrigel transwell culture plates with 4 μm pore size inserts were used. After cultured with HG, 2 × 10^5^ cells were loaded onto the upper chambers of transwell culture plates with FBS-free ECM, which were suspended over the lower wells with 5% FBS-containing ECM. After 24 h incubation, migrated cells in the upper chambers were stained with 1% crystal violet and then counted.

### Statistical analysis

All experiments were conducted at least thrice. Data are expressed as the mean ± standard deviation (SD). Differences between the treatment groups were analyzed by one-way ANOVA followed by Tukey’s post hoc test and Student’s *t* test using Prism (version 5, GraphPad). A value *P* < 0.05 was considered statistically significant.

## Results

### Reversal of HG-induced mitochondrial fission and dysfunction in HRGECs by S1PR2 antagonist

**Fig. 1:**
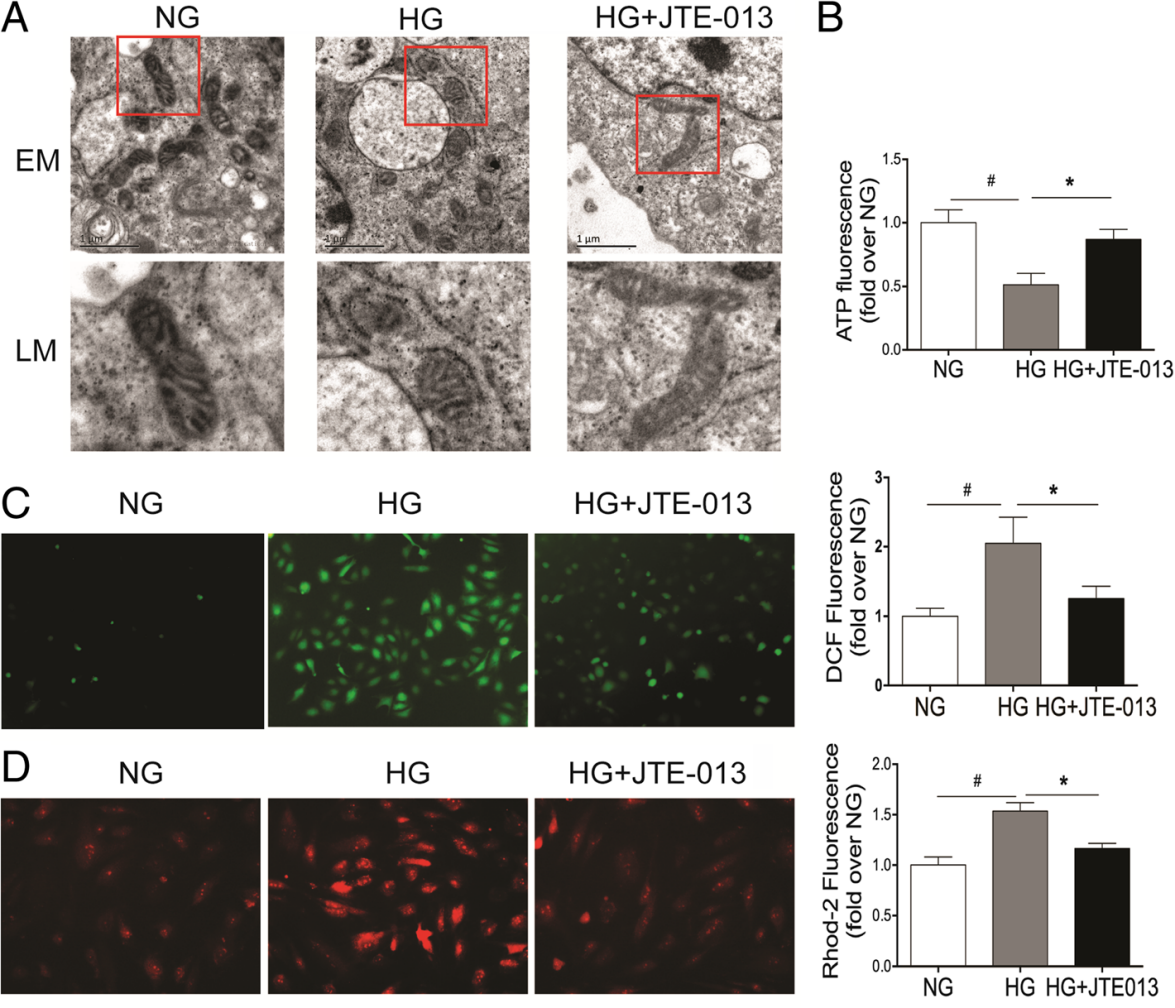
Inhibition of S1PR2 reverses HG-induced mitochondrial fission and dysfunction. HEGECs were pretreated with JTE-013, a S1PR2 inhibitor, for 30 min and then incubated with normal glucose (NG, 5.5 mM) or high glucose (HG, 30 mM) for 72 h. **a** Mitochondrial morphology changes were observed under an electron microscopy. **b** ATP production was measured using a commercial ATPlite assay kit and quantified by reading ATP luminescence on a microplate reader. **c** Total intracellular ROS generation was assessed by using the fluorogenic probe DCF-DA and observed under a fluorescent microscope, and further quantified with flow cytometric assay. **d** Ca^2+^ levels in mitochondria were determined by using the Ca^2+^ probe Rhod-2 AM and imaged with a fluorescent microscope, and quantified with flow cytometry. Data were normalized with the values of the NG-treated cells set as 1. Results are expressed as mean ± SD of three independent experiments. *P < 0.05 versus the HG group; ^#^P < 0.05 versus the NG group. EM, electron microscopy; LM, local magnification

To determine the regulatory role of S1PR2 in mitochondria, we used HG to induce mitochondrial morphological and metabolic changes. HRGECs were pretreated with the S1PR2 inhibitor JTE-013 and then incubated with HG. We found that S1PR2 antagonist ameliorated the elevated mitochondrial fragmentation, indicated by the appearance of small and punctate mitochondria (Fig. 1a). Mitochondrial ROS generation, calcium overload, and ATP reduction induced by HG were reversed by JTE-013 as well (*P* < 0.05) (Fig. 1b-d). Since Mnt treatment did not produce these biochemical and physiological changes, the osmotic effect of HG could be ruled out (Additional file 1: Figure S1).

### Reversal of HG-induced dysfunction of HRGECs by S1PR2 antagonist

Moreover, the increased levels of cell permeability and apoptosis were markedly reduced and the level of migration was significantly elevated in the HG + JTE-013 group compared to the HG-treated group.

**Fig. 2:**
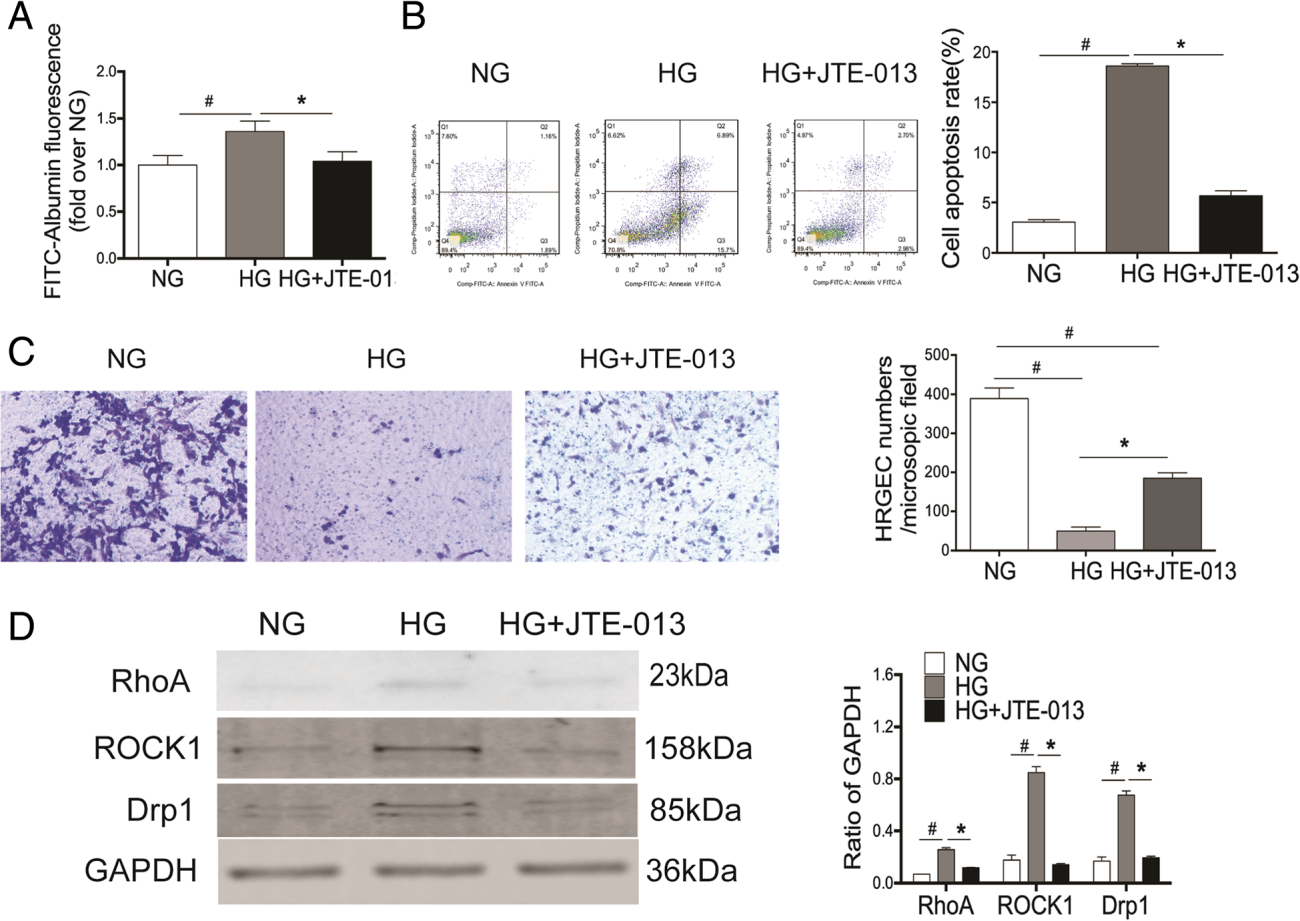
Inhibition of S1PR2 reverses HG-induced endothelial cell dysfunction. HEGECs were treated as described in Fig. 1. **a** The cell permeability was assessed by measuring the FITC-BSA that crossed the monolayer, and quantified by a fluorescence plate reader. **b** HRGECs were stained with both Annexin V and propidium iodide (PI), then determined using flow cytometric analysis. **c** The degree of migration of different groups was quantified by microscope. **d** The expression levels of RhoA, ROCK1, and Drp1 were evaluated by Western blot analysis. The band densities of these proteins were quantified with GAPDH as a loading control. Results are expressed as mean ± SD of three independent experiments. *P < 0.05 versus the HG group; ^#^P < 0.05 versus the NG group

HG increased cell permeability, induced cell apoptosis, and inhibited cell migration. However, the pretreatment of cells with JTE-013 significantly alleviated these HG-mediated effects (*P* < 0.05) (Fig. 2a-c). Similarly, the osmotic effect of HG was not involved in these biochemical and physiological changes since Mnt effect was not seen (Additional file 1: Figure S2). These results suggested that S1PR2 may play a potential role in HG-induced detrimental effects on mitochondrial morphology and function.

Since ROCK1 has been reported to be activated by S1PR2 and to promote Drp1 recruitment to mitochondria during HG-induced mitochondrial fission, the responses of ROCK1 and Drp1 protein expression to HG stimulation were also observed. We found that S1PR2 antagonist resulted in decreased protein expressions levels of RhoA, ROCK1 and Drp1 in the HG + JTE-013 group compared with the HG group (P < 0.05) (Fig. 2d), indicating that S1PR2 works upstream of RhoA, ROCK1, and Drp1and regulates their protein expression in HRGECs. Collectively, these data indicate that S1PR2 is critical in the remodeling of mitochondrial morphology and mitochondrial functions, and may participate in hyperglycemia-induced dysfunction of HRGECs, possibly by regulating RhoA/ROCK1.

### Reversal of HG-induced mitochondrial fission and dysfunction by inhibition of ROCK1

**Fig. 3:**
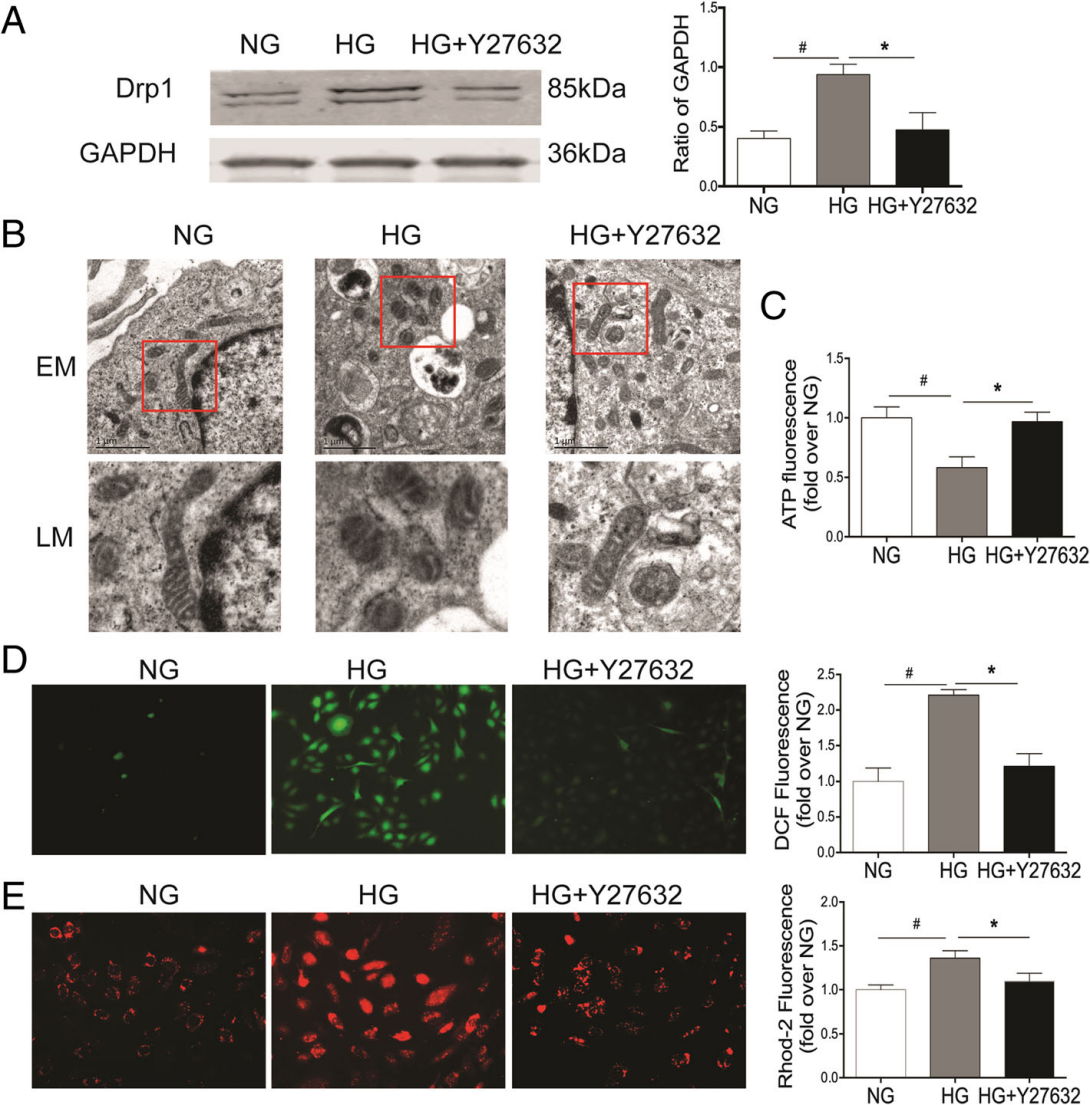
Inhibition of ROCK1 reverses HG-induced mitochondrial fission and dysfunction. HEGECs were pretreated with the ROCK1 inhibitor Y27632 for 30 min and then incubated with normal glucose (NG) or high glucose (HG) for 72 h. Drp1 protein expression (**a**), mitochondrial morphology (**b**), intracellular ATP production (**c**), ROS generation (**d**), and Ca2+ concentration (**e**) were assessed as described in Fig. 1. Results are expressed as mean ± SD of three independent experiments. *P < 0.05 versus the HG group; ^#^P < 0.05 versus the NG group. EM, electron microscopy; LM, local magnification

RhoA/ROCK1 is the key signaling pathway that couples S1PR2 and modulates endothelial cell dysfunction induced by hyperglycemia. However, the role of ROCK1 associated with S1PR2 in mitochondrial fission and dysfunction remains unclear. To elucidate the role of ROCK1 in mitochondrial fission and dysfunction induced by HG, HRGECs were pretreated with the ROCK1 inhibitor Y27632 for 30 min and then incubated with HG for 72 h. Since Drp1 is the key regulator of the mitochondrial fission machinery, we therefore tested whether ROCK1 mediates mitochondrial fission and dysfunction through the regulation of Drp1. As shown in Fig. 3a, HG-induced Drp1 expression was significantly blunted when Y27632 was present in the culture medium (*P* < 0.05), indicating ROCK1 is required for the upregulation of Drp1 expression upon HG stimulation. Our further studies demonstrated that the inhibition of ROCK1 by Y2763 improved HG-induced effects on mitochondrial fragmentation, ATP production, ROS generation, and Ca^2+^ hemostasis (P < 0.05) (Fig. 3b-e).

### Reversal of HG-induced cell dysfunction by inhibition of ROCK1

**Fig. 4:**
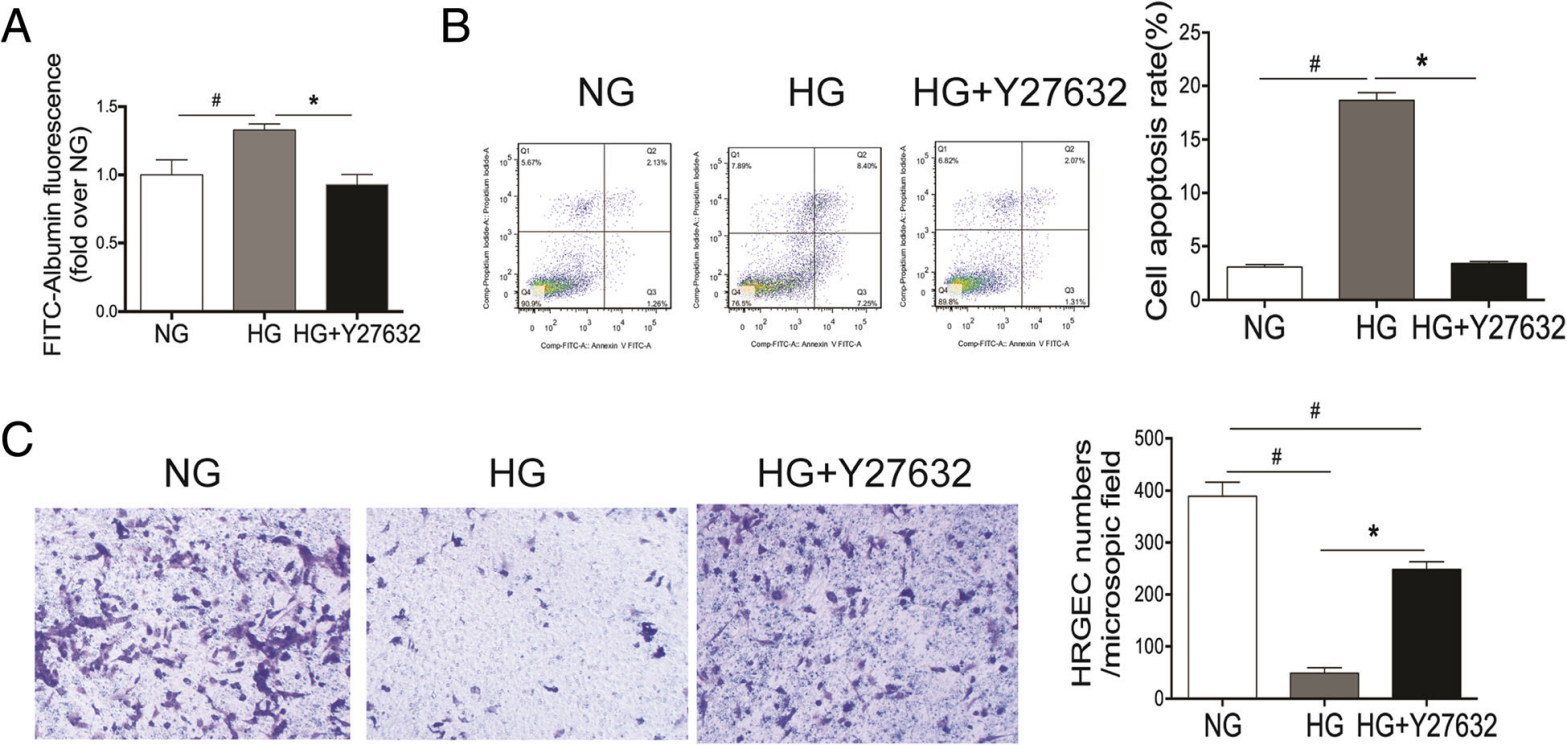
Inhibition of ROCK1 reverses HG-induced endothelial cell dysfunctions. HEGECs were pretreated with the ROCK1 inhibitor Y27632 for 30 min and then incubated with normal glucose (NG) or high glucose (HG) for 72 h. Cell permeability (**a**), apoptosis (**b**), and migration (**c**) were assessed as described in Fig. 3. Results are expressed as mean ± SD of three independent experiments. *P < 0.05 versus the HG group; ^#^P < 0.05 versus the NG group

As the first barrier of glomerular filtration membrane, maintaining an intact cell junction is the core function of HRGECs. We found that inhibition of ROCK1 suppressed the increased cell permeability induced by HG (P < 0.05) (Fig. 4a**)**, which may contribute to the break of glomerular filtration membrane. Moreover, increased apoptosis and the reduced migration of cells by HG were significantly suppressed by Y27632 (P < 0.05) (Fig. 4b-c). These results suggest that ROCK1 play a crucial role in hyperglycemia-induced dysfunction of HRGECs, at least partially through the regulation of Drp1 expression and activity.

## Discussion

In this study, we explored the role of S1PR2 and its downstream signal molecules in the morphology and function of mitochondria in primary HRGECs. Our results demonstrated that the S1PR2 antagonist (JTE-013) almost completely blocked endothelial dysfunction and associated fission and dysfunction of mitochondria under HG conditions along with down-regulated HG-promoted expression of RhoA/ROCK1. Our data also indicate that ROCK1 is a critical step that links S1PR2 and mitochondrial fission and dysfunction through regulating Drp1. Therefore, we collected direct evidence for the mechanistic association between S1PR2 and cell dysfunction via the activation of the RhoA/ROCK1/Drp1 signaling pathway in HRGECs.

Vascular complications are the initial factor of mortality associated with type 2 diabetes mellitus [27]. Diabetic nephropathy, a microvascular complication,, is the most common cause of end-stage renal disease in developed countries [28]. Extensive studies have confirmed that vascular diseases usually start with endothelial cell damage [29]. We used the primary HRGECs for the first time to explore the potentially pathological role of S1PR2 in the dysfunction of primary renal endothelium cells. We had previously observed that S1PR2 displays a positive regulation of human umbilical vein endothelial cell dysfunctions induced by hyperglycemia [30]. Here, we further demonstrated that S1PR2 is important for regulating HG-induced dysfunction in HRGECs, as indicated by the use of JTE-013, which reversed the inducing effects of HG on the barrier function, migration, and cell death of HRGECs. Small GTPases (Rho, Rac and Ras) are known downstream signaling effectors of S1PR2 [6–8]. We confirmed that S1PR2 inhibition blocks HG-induced upregulation of the expression of the small GTPase RhoA and its downstream ROCK1, indicating that S1PR2 regulates the dysfunctions of primary HRGECs through activating the RhoA/ROCK1 pathway.

Although mitochondria represent only 5–10% of cytoplasmic volume of endothelial cells [31], they are the most important organelles for cell survival [32]. Balanced mitochondria dynamics, ATP production, ROS synthesis, and local Ca^2+^ concentrations are all indispensable for healthy endothelial function [14, 33]. The present study shows the reversal of HG-mediated mitochondria fission and dysfunction by S1PR2 inhibition, suggesting that S1PR2 is responsible for the regulation of endothelial mitochondria morphology and function. Previous work has shown that the balance between mitochondrial fission and fusion, in which Drp1is a crucial player, holds the key to maintain mitochondrial morphology and function [34, 35]. As the RhoA/ROCK1 is the downstream pathway of S1PR2 in HRGECs and Drp1 a direct substrate of ROCK1 in podocytes contributing to the enhanced mitochondrial fission [36], we hypothesized that ROCK1 is the key regulator linking S1PR2 and mitochondrial fission in HRGECs. As expected, inhibition of ROCK1 downregulated the expression of Drp1 and consequently improved HG-induced mitochondrial morphology and biochemical changes in HRGECs.

## Conclusions

Overall, our study provides the first evidence that S1PR2 exerts multiple effects on the fission and dysfunction of mitochondria induced by HG in HRGECs, which in turn contribute to endothelial dysfunction. Additionally, ROCK1 is a key regulator that functionally links S1PR2 and mitochondrial fission and dysfunction through activating Drp1. Although the exact mechanisms behind the ROCK1/Drp1 regulation by S1PR2 remind further investigation, we support the concept of S1PR2-mediated morphology and function of mitochondria of HRGECs induced by HG through the RhoA/ROCK1/Drp1 signaling pathway, suggesting that targeting this S1PR2-associated signaling pathway might be a potential therapeutic target for renal vascular disease treatment.

## Additional file

Additional file 1: **Figure S1.** Mitochondrial morphology and physiological functions in HRGECs treated with normal glucose (NG, 5.5 mM), high glucose (HG, 30mM), or mannitol (Mnt, 30mM) as an osmotic glucose (NG, 5.5 mM), high glucose (HG, 30mM), or mannitol (Mnt, 30mM) as an osmotic control for 72 h. (A) Mitochondrial morphology changes were observed under an electron microscopy. (B) ATP production was measured using a commercial ATPlite assay kit and quantified by reading luminescence on a microplate reader. (C) Total intracellular ROS generation was assessed by using the fluorogenic probe DCF-DA and observed under a fluorescent microscope, and further quantified with flow cytometric assay. (D) Ca^2+^ levels in mitochondria were determined by using the Ca^2+^ probe Rhod-2 AM and imaged with a fluorescent microscope, and quantified with flow cytometry. Data were normalized with the values of the NG-treated cells set as 100% or 1. Results are expressed as mean ± SD of three independent experiments. *P < 0.05 versus the NG group; #*P* < 0.05 versus the Mnt group. **Figure S2**. S1PR2 antagonist reverses HG-induced endothelial cell dysfunction. HEGECs were pretreated with JTE-013, a S1PR2 inhibitor, for 30 min and then incubated with normal glucose (NG) or high glucose (HG) for 72 h. (A) The cell permeability was measured the FITC-BSA that crossed the monolayer, and quantified by fluorescence plate reader. (B) HRGECs were stained with both Annexin V and propidium iodide (PI), then determined using flow cytometric analysis. (C) The degree of migration of different groups was quantified by microscope. Results are expressed as mean ± SD of three independent experiments. *P < 0.05 versus the HG group; #*P* <0.05 versus the NG group. (DOCX 9720 kb)

## Abbreviations

DCF-DA: 2′,7′- dichlorofluorescein diacetate
Drp1: Dynmin-related protein-1
ECM: Endothelial Cell Medium
HG: High glucose
HRGECs: Human renal glomerular endothelial cells
Mnt: Mannitol
NG: Normal glucose
PI: Propidium iodide
ROCK1: Rho-associated coiled coil-containing protein kinase 1
RT: Room temperature
S1PR2: Sphingosine-1-phosphate receptor 2
SD: Standard deviation
