# Supplementary material for: S1PR2 antagonist ameliorate high glucose-induced fission and dysfunction of mitochondria in HRGECs via regulating ROCK1
Source: BMC Nephrol. 2019 Apr 18;20:135. doi: 10.1186/s12882-019-1323-0 (PMC6471837; doi:10.1186/s12882-019-1323-0)
Supplement: Supplementary file 1 — Figure S1. Mitochondrial morphology and physiological functions in HRGECs treated with normal glucose (NG, 5.5 mM), high glucose (HG, 30mM), or mannitol (Mnt, 30mM) as an osmotic glucose (NG, 5.5 mM), high glucose (HG, 30mM), or mannitol (Mnt, 30mM) as an osmotic control for 72 h. (A) Mitochondrial morphology changes were observed under an electron microscopy. (B) ATP production was measured using a commercial ATPlite assay kit and quantified by reading luminescence on a microplate reader. (C) Total intracellular ROS generation was assessed by using the fluorogenic probe DCF-DA and observed under a fluorescent microscope, and further quantified with flow cytometric assay. (D) Ca2+ levels in mitochondria were determined by using the Ca2+ probe Rhod-2 AM and imaged with a fluorescent microscope, and quantified with flow cytometry. Data were normalized with the values of the NG-treated cells set as 100% or 1. Results are expressed as mean ± SD of three independent experiments. *P < 0.05 versus the NG group; #P < 0.05 versus the Mnt group. Figure S2. S1PR2 antagonist reverses HG-induced endothelial cell dysfunction. HEGECs were pretreated with JTE-013, a S1PR2 inhibitor, for 30 min and then incubated with normal glucose (NG) or high glucose (HG) for 72 h. (A) The cell permeability was measured the FITC-BSA that crossed the monolayer, and quantified by fluorescence plate reader. (B) HRGECs were stained with both Annexin V and propidium iodide (PI), then determined using flow cytometric analysis. (C) The degree of migration of different groups was quantified by microscope. Results are expressed as mean ± SD of three independent experiments. *P < 0.05 versus the HG group; #P <0.05 versus the NG group. (DOCX 9720 kb) [file 12882_2019_1323_MOESM1_ESM.docx]

**
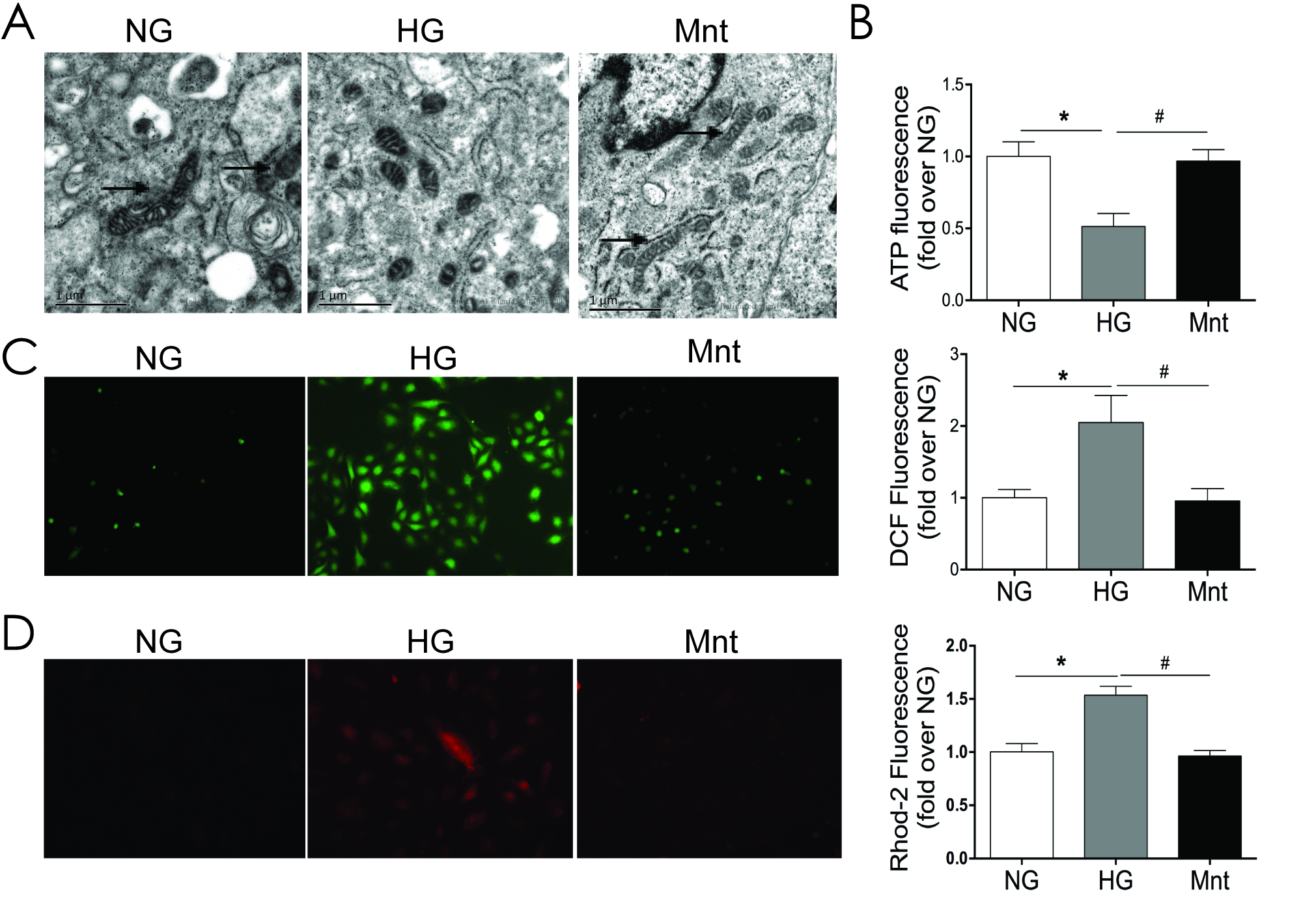
**

Fig S1. Mitochondrial morphology and physiological functions in HRGECs treated with normal glucose (NG, 5.5 mM), high glucose (HG, 30mM), or mannitol (Mnt, 30mM) as an osmotic control for 72 h. (A) Mitochondrial morphology changes were observed under an electron microscopy. (B) ATP production was measured using a commercial ATPlite assay kit and quantified by reading luminescence on a microplate reader. (C) Total intracellular ROS generation was assessed by using the fluorogenic probe DCF-DA and observed under a fluorescent microscope, and further quantified with flow cytometric assay. (D) Ca^2+^ levels in mitochondria were determined by using the Ca^2+^ probe Rhod-2 AM and imaged with a fluorescent microscope, and quantified with flow cytometry. Data were normalized with the values of the NG-treated cells set as 100% or 1. Results are expressed as mean ± SD of three independent experiments. *P < 0.05 versus the NG group; ^#^P < 0.05 versus the Mnt group.

**
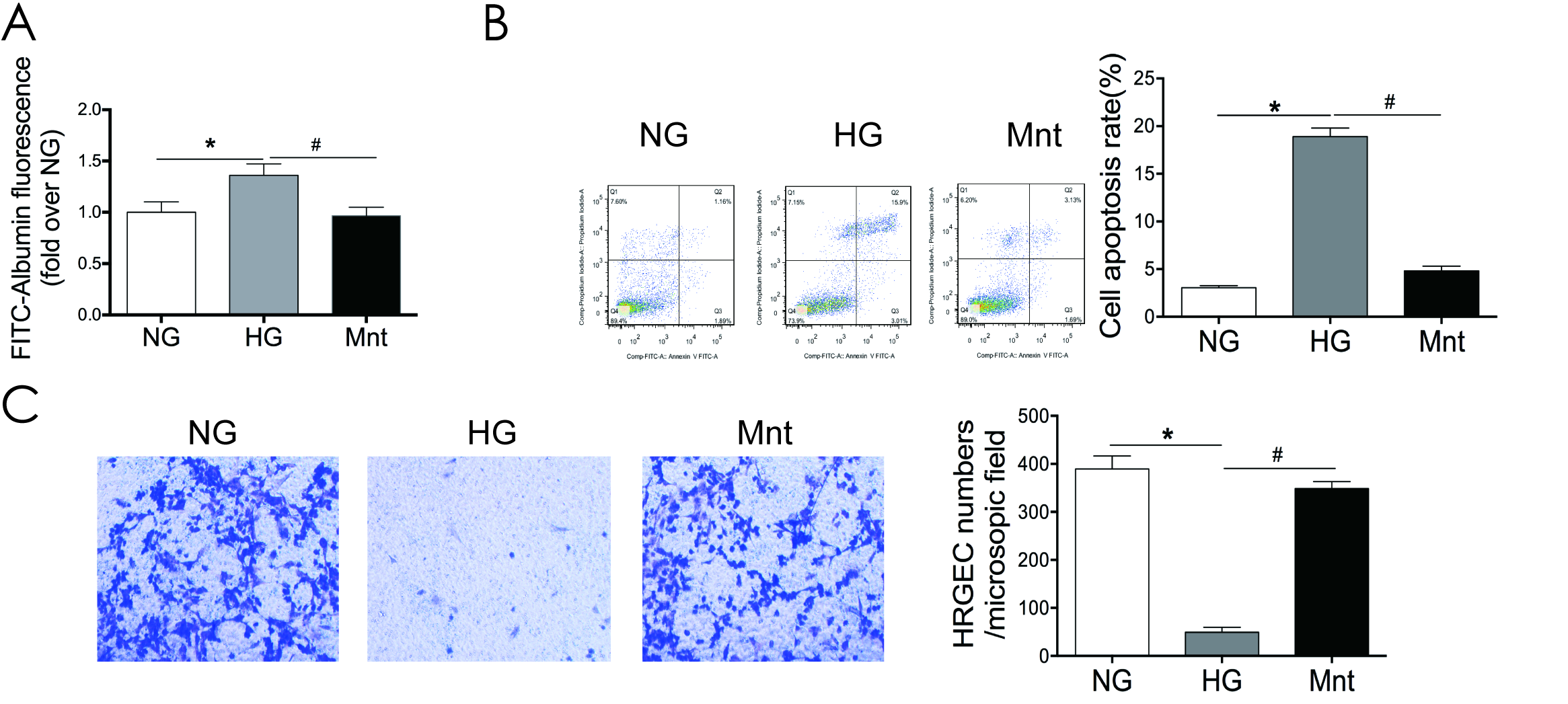
**

Fig S2. S1PR2 antagonist reverses HG-induced endothelial cell dysfunction. HEGECs were pretreated with JTE-013, a S1PR2 inhibitor, for 30 min and then incubated with normal glucose (NG) or high glucose (HG) for 72 h. (A) The cell permeability was measured the FITC-BSA that crossed the monolayer, and quantified by fluorescence plate reader. (B) HRGECs were stained with both Annexin V and propidium iodide (PI), then determined using flow cytometric analysis. (C) The degree of migration of different groups was quantified by microscope. Results are expressed as mean ± SD of three independent experiments. *P < 0.05 versus the HG group; ^#^P < 0.05 versus the NG group.
